# An augmented reality interface to control a collaborative robot in rehab: A preliminary usability evaluation

**DOI:** 10.3389/fdgth.2023.1078511

**Published:** 2023-02-13

**Authors:** José Carlos Rodrigues, Paulo Menezes, Maria Teresa Restivo

**Affiliations:** ^1^ LAETA-INEGI, Faculty of Engineering, University of Porto, Porto, Portugal; ^2^ Department of Electrical and Computer Engineering, Institute of Systems and Robotics, University of Coimbra, Coimbra, Portugal; ^3^ LAETA-INEGI, University of Porto, A3ES, Porto, Portugal

**Keywords:** augmented realitiy, collaborative robot, user experence, upper-limb rehabilitation, serious games, system usability scale (SUS), user experience questionnaire (UEQ), flow short scale (FSS)

## Abstract

Human emotions can be seen as a valuable variable to explore in Human-Computer Interaction for effective, efficient, and satisfying interface development. The inclusion of appropriate emotional triggers in the design of interactive systems can play a decisive role in users' acceptance or rejection. It is well known that the major problem in motor rehabilitation is the high dropout rate resulting from the frustrated expectations given the typical slow recovery process and consequent lack of motivation to endure. This work proposes grouping a collaborative robot with one specific augmented reality equipment to create a rehabilitation system where some gamification levels might be added to provide a better and more motivating experience to patients. Such a system, as a whole, is customizable to adapt to each patient's needs on the rehabilitation exercises. By transforming a tedious exercise into a game, we expect to create an additional layer of enjoyment that can help in triggering positive emotions and stimulate users to continue the rehabilitation process. A pre-prototype was developed to validate this system's usability, and a cross-sectional study using a non-probabilistic sample of 31 individuals is presented and discussed. This study included the application of three standard questionnaires on usability and user experience. The analyses of these questionnaires show that the majority of the users found the system easy and enjoyable. The system was also analysed by a rehabilitation expert who gave a positive output regarding its usefulness, and positive impact on its use in the upper-limb rehabilitation processes. These results clearly encourage further development of the proposed system.

## 1. Introduction

In Human-Computer Interaction (HCI), emotions can be seen as the variables to explore for effective, efficient, and satisfying interface development. The reason is that affective and emotional states are involved in activities where individuals are engaged, including tasks performed in front of a computer or interacting with computer-based technology. For example, understanding a user's emotional state induced when receiving a piece of information can be precious. Was the user happy, increasingly confused, frustrated, or amused? Did that emotional state change upon receiving the information (or stimulus)?

To this end, immersive systems can help provide multiple stimuli to the user, particularly auditory and visual, but that can be complemented with other ones, e.g., heat radiation, cold breeze, or haptic stimuli, when coupled with external hardware for that purpose.

The interaction design of an immersive environment can be the key to its motivational success if the correct emotional triggers are included. Immersive technologies have had a significant improvement over the last few years, along with increased computational power and higher graphical quality levels. Therefore, new levels of immersion and natural interaction are becoming possible when compared to previous devices.

During the process of emotional activation, individuals tend firstly to evaluate a stimulus as “good” or “bad” (1, 2), including the evaluation of the event as interfering with or facilitating the achievement of the individual's goals (3). This initial evaluation is immediate, unconscious, and automatic (1) and rapidly motivates a behavioural response of avoidance or approximation to the stimulus source depending on the evaluated outcomes (4). Emotions are, therefore, directly linked to motivation (5).

In motor rehabilitation, motivation is on par with adherence as the factor for a successful therapeutic process. Extensive repetition of the same task can lead to an emotional state of boredom, decreasing patient motivation and adherence to performing the designated exercises, which can be potential barriers to recovery (6, 7). Since a systematic repetition of the same routine is vital for improving the motor functions of patients, it is essential to provide a therapeutic environment that can help improve patient motivation (8–10). This is critical for patient retention during the necessary period for the treatment process to achieve the best possible outcome. To this end, game design principles can help create an additional layer of enjoyment by creating a challenging and rewarding environment that can help stimulate users and trigger positive emotions that, in consequence, promote positive feelings and therefore increase their motivation to perform the therapy exercises.

According to Viglialoro et al. (11), clinical trials using immersive environments have shown benefits when considering the patients' usability, entertainment, and motivation compared to traditional rehabilitation therapy methods. Immersive environments cannot replace standard therapy, but they enrich the user experience and, therefore, their motivation and adherence to treatment. As mentioned above, immersive environments based on head-mounted displays (HMD) can only provide auditory or visual stimuli, and adding other types of stimuli implies using complementary devices or hardware. Robotic systems can be used to this end as they enable tactile/force feedback to the user while supporting the controlled motion of a patient's limb during exercises.

Furthermore, in rehabilitation, these can play an essential role in maintaining and monitoring the users' movement quality, as these are also crucial in the treatment outcome. As a result, Robot-assisted therapy is an emerging practice in treating motor functions, mainly for the upper-limbs, which can help users achieve repetitive high-intensity training while maintaining movement quality (12). The usage of robots can also reduce the need for constant and direct supervision by therapists since they can automate part of the process and monitor the speed and accuracy of the users' movements (13). Collaborative robots are a small class of emerging robots capable of working with humans and minimizing the risk of high-velocity impacts or injuries by collisions (14), instead of traditional industrial robots, which are not recommended to work in a shared space with humans (15). Additionally, robots in therapy may allow performance-based strategies to monitor the patients' progress over time (16–18).

Moreover, Augmented Reality (AR) and Mixed Reality (MR) systems, in particular, modify the perception of the physical world *via* an additional virtual layer, adding extra information in the form of images, videos, text, or other virtual objects to the existing physical environment. Azuma et al. (19) define AR as a system that “supplements the real world with virtual (computer-generated) objects that appear to coexist in the same space as the real world.” MR systems, on the other hand, enable the interaction between the physical and virtual contents. Parveau et al. (20) define it as “the ability to interact with both physical and virtual objects which are registered in time and according to the user's environment.” The connection between the physical and virtual worlds allows the creation of an additional layer of visual and auditory feedback. These combined with tactile feedback provided by the robotic arm can be used to create new experiences for the users by using gamification principles, such as new challenging scenarios, reward systems and even the perspective of overcoming his/her higher score. Therefore, these extrinsic motivation aspects can help retain or promote engagement of users during the rehabilitation process.

This work is focused on the preliminary analysis of the usability of a system that integrates augmented reality and a collaborative robot aiming for its use as a therapeutic support tool. This usability evaluation was done to validate the core idea that using these systems does not create a hindrance. Instead, it can help users in performing tasks easier and help them to stay motivated.

### 1.1. State of the art

Several studies have used robotics to help the limb rehabilitation process. However, most of these examples are with robots explicitly designed for that task, and usually, they act as passive or active therapy. Passive treatment involves moving the impaired limb in a preplanned trajectory many times during a session, which can be performed with the help of an exoskeleton robot (21). This kind of treatment focuses on abduction and adduction of the impaired limb (22), which can also be used to assess the range of motion (23). Ren et al. (24) performed a clinical study that showed effectiveness in reducing the spasms and stiffness of the impaired limbs with passive therapy. On another side, active therapy is prescribed for patients who can move their impaired limbs to some limits. The active term refers to exploiting the patient's ability to move the impaired limb to recover some of the lost functionality (25). Active therapy can be classified as active-assistive therapy or active-resistive therapy. Active-assistive therapy involves applying an external force by a therapist or robot to help the patient fulfil the appointed task (26). It also improves the patient's range of motion (27). Active-resistive treatment involves applying an opposing force on the impaired limbs. A therapist or robot can apply the opposite force (24). Hu et al. (28) and Fasoli et al. (29) showed that the patient's performance improved gradually when using incremental opposing forces to their movement.

For the purpose of active-assistive therapy, MIT-MANUS (30), a robotic workstation developed by the Massachusetts Institute of Technology, is used to rehabilitate upper-limbs post-stroke. It is an interactive station where the patient interacts with a PC game to follow specific movements visually. The workstation provides five degrees of freedom, two for the elbow and forearm and three for the wrist. It has been experimentally proven to provide positive therapeutic effects for upper-limb impairment (31).

Lum et al. (32) describe a Mirror Image Movement Enabler (MIME), a continuous passive movement robotic system consisting of a wheelchair and a height-adjustable table. The patient sits in the wheelchair and places the affected limb on the adjustable table. The limb is then strapped into the forearm split, which restricts wrist and hand movements. The robotic system can then operate in passive mode or active-resistive mode. The robot moves the limb in a specific trajectory towards the target in the passive mode.

In contrast, the active-resistive method provides resistance to the user movement in the identified course, and the patient has to provide maximal effort to reach the target. This system strengthens the muscles and improves the limb's motions. One other example of a continuous passive movement robotic system is described by Reinkensmeyer et al. (33) as an automated system with one degree of freedom to train and evaluate upper-limb functions. The system uses the reaching principled therapy technique, where the patient's arm is attached to a splint, and the patient is advised to reach for objects. The system's orientation can be changed manually between the horizontal and vertical planes.

ARMin (34) is an upper-limb rehabilitation robot that provides seven degrees of freedom, allowing complete control of the patient's arm movement from shoulder to hand. The patient attaches their hand to the robotic arm and adjusts its length while sitting in a chair with the robotic arm. Results showed an improvement in the limb's motion, where the user could extend the limbs to further distances. In addition, the strength of the support decreased gradually as the patient recovered their motor capacities.

While robotics in rehabilitation has been an established field of research, most robotic systems were designed with a specific objective. In recent years, the possibility of using emerging collaborative robots is emerging as a new research area. Collaborative robots are an emerging class of industrial robots designed to be used in conjunction with humans without safety risks.

Kyrkjebø et al. (14) work discuss the possibility of using a commercial collaborative robot (UR5e) as a motor rehabilitation tool for the upper-limbs. Safety and control aspects are analyzed and discussed to determine the feasibility of using standard industrial collaborative robots for rehabilitation. Kyrkjebø et al. conclude that using the industrial collaborative robot as a rehabilitation tool is feasible when combined with accurate force and torque measurements since the robot allows customizing a wide range of movements to be used in the treatment processes.

Azevedo Fernandes et al. (35) study addresses using a collaborative robot applied in the rehabilitation field to help upper-limb physiotherapy, specifically shoulder rehabilitation. The work proposes a system capable of learning from patient usage to create motion paths to perform the exercises. A reinforcement learning algorithm was used to make the system robust and independent of the motion path. The main contribution of this work is the possibility of testing the system with a model of human contact before its application. Besides, inserting a self-control module removes the need for the robot's path planning and configuration for each patient. In this case, the dynamic simulation can provide an excellent gain for therapists because it helps them learn the proposed system. It also allows the therapists to test new methods in the simulation environment.

Chiriatti et al. (36) work proposes a methodology as a starting point for the study of the integration of collaborative robots into rehabilitation practices and evaluating its feasibility. This work presents a general framework for studying the kinematics and dynamics of a human-robot system designed to rehabilitate the upper arm. Kinematic and dynamic models were developed to assess the feasibility of the UR5 robot. It is concluded that the forces and moments at the human-robot interface are tolerable and suitable for the rehabilitation procedure aimed at improving human strength.

Liberatore et al. (37) systematic review provides significant insight into immersive systems research where two research areas stand out in their study—training and rehabilitation. Training in assembling or performing maintenance tasks and rehabilitation exercises can be a slow and demotivating process that heavily relies on repetition-based training. As shown in (37), immersive systems can help users' motivation and adherence to repetition-based training. However, it can also help the users achieve a better performance than control groups that do not use immersive systems. In recent years, there have been increasing studies regarding the benefits of using immersive environments in therapy. The patient's motivation and performance can be positively impacted compared to a control group that performs the same exercises without using these systems (38). Lin et al. (39) describe the benefits of using Virtual Reality (VR) to improve patients' motivation to recover from a stroke while engaging in home-based therapy. Given that daily usage and the high number of repetitions are critical aspects for a better treatment outcome, this VR application was identified as potentially increasing the effectiveness of therapy service when conducted at home. Also, Levin (10) recognizes the need to create some intense repetition-based activities for motor recovery, and VR is identified as an effective tool for designing those environments. VR and AR applications can also have the capability to automatically record and objectively evaluate the user's performance, which is particularly important in the rehabilitation field (25, 40) since traditional rehabilitation methods are based on subjective progress evaluation, and they lack objective performance goals. However, AR can be an even better tool in motor rehabilitation since AR supplements reality but does not replace it. It can provide the user with a better sense of presence and reality judgment of the environment. It can also preserve the possibility of interacting directly with real instrumentation and other subjects, such as therapists (41). According to a recent literature review (11), the first clinical studies show clear benefits of AR-based rehabilitation over traditional methods regarding usability, enjoyability, user motivation, and improving patient performance outcomes. Even if additional clinical studies are needed to generalize these findings, the results encourage further investigations and technical development. Also, this study shows that using an HMD in augmented reality applications has not been fully explored in rehabilitation. Most studies include spatial displays (screen or projection-based displays) and hand-held displays (smartphones or tablets).

Another critical aspect during the therapy sessions is maintaining the patients' motivation and engagement in doing the recommended repeatedly boring rehabilitation exercises. Low motivation and adherence are a barrier to the potential recovery of the patients (6, 7). Since repetitive and high-dosage rehabilitation exercises can improve the patient's motor abilities (7–9), it is crucial to provide an engaging environment to help to increase the users' motivation. In this perspective, using gaming rules can help achieving higher patient motivation.

When gaming rules are applied in any sector unrelated to gaming, it is designated by gamification (42). Gamification in healthcare is a patient engagement technique that aims to generate entertainment for the patients. Using game-design elements in the healthcare context can add significant value to the rehabilitation process. They can create diverse and interactive environments that can enhance patient engagement, socialization, feedback, and adherence to the treatment process and to provide better health outcomes (43–45). In other areas like e-commerce or marketing, gamification methods have proven to enhance co-creation value. Customers engaged in these methods are more willing to make use of the products (46). Gamified methods should be inspired by the PERMA model (47). This model represents the five core elements of happiness and well-being: positive emotion, engagement, relationships, meaning, and accomplishments. Providing small goals, rewards, and visual or audio feedback is noted that plays a significant role in the patients' engagement. In turn, this allows for the patients to know the improvement in their health each time they play (48).

The measurement quality of the executed movement while performing the therapy exercise also provides an objective evaluation and therefore can be seen as a possible motivational factor in the rehabilitation process and how well the patients can recover from their impairments. This movement quality can be seen into three components considered essential in upper-limb rehabilitation process: (i) speed, (ii) exercise path accuracy, (iii) posture (49).

## 2. Materials and methods

### 2.1. Goals and approach

This paper explores and evaluates the usability of a system that integrates a collaborative robot added by augmented reality technology for rehabilitation. The system under development uses a UR5e and Microsoft HoloLens 2 (MH_2). The choice for the utilisation of the UR5e relied on several factors: (1) it supports a load of 5Kg, which allows the robot to work as an active or passive rehabilitation system; (2) the robotic arm's 85 cm reach enables a good movement amplitude for upper-limb rehabilitation; (3) it is on the market and studies suggest it is an adequate and safe tool to be used in upper-limb rehabilitation (14, 35, 36), and (4) the UR5e was readily available in the lab for the pre-prototype. Additionally, the robot will collect all the data during the exercise and create an adapting haptic stimulation according to the evolution of the rehabilitation process. On the other hand, the MH_2 provides an augmented reality interface allowing the user to access a set of games that can enhance his/her motivation.

By combining these two types of hardware, we aim to create a system capable of being used in rehabilitation as a tool to promote positive emotions and capable of operating autonomously, precisely, and objectively. Therefore, factors like motivation and adherence to the treatment, the quality of the movement achieved, and their high repetition are critical aspects of successful treatment outcomes.

The integration of a virtual interface with a robotic arm will allow the therapist to choose the type of exercise for the patient's needs; customize the exercise parameters according to the patient's capabilities; introduce convenient anthropometric data; define settings as the number of repetitions, velocity, range, etc. according to each patient's stage; record and display data of patients' performance and evolution. From the patient's point of view, the system offers customized training; visualization of the motion path to guide him/her; a set of engaging serious games to promote motivation and mitigate the lack of human-robot emotional interaction; and an intelligent algorithm that can either help (in early stages) or offer resistance (in advanced rehabilitation stages) to the movements.

The use of serious games was considered as enabling out-of-ordinary context that patients are used to, which can help motivate them. This is especially beneficial in neurological cases since high repetition of the correct movement supports a cortical integration of the neural pathways. The more realistic the virtual environment provided by the system is, the higher the neural activation will be.

To evaluate the system's (HoloLens and UR5e) interface usability, a pre-prototype was developed, and a case study with 31 participants of a wide range of ages was conducted. For this usability evaluation of the pre-prototype, the used sample size is very good.According with J. Nielsen (50) for a small or medium project, detection of early problems in usability of a system a small sample of users is needed to detect most of the problems on an initial version of any system. Also according with the same author, by using the following formula $N(1-{\left(1-\lambda \right)}^{n})$, where N is the total number of usability problems in the design, and λ the proportion of usability problems discovered while testing a single user (which has a typical mean value of 31%), and n is the number of test users. The probability of finding a usability problem when using 5 users is approximately 75%, with 7 users is approximately 93%, and with 15 users is approximately 99.6%. A basic serious game was implemented in this pre-prototype. The game's main objective was to have the users control the robotic arm by applying force on its free handle and moving it like a joystick. Through the augmented reality interface, the user visualizes the motion path to perform and the scoring system as a stimulus to overcome the displayed highest score.

However, before testing the initial development of the pre-prototype, the authors were conscient of the need to understand better what type of exercises are clinically correct, not only to avoid incorrect movements but also to identify the elementary upper-limb movements for a correct rehabilitation procedure (an incorrect movement type can be harmful instead). In fact, the User-Driven Design (UDD) is a methodology that is mandatory to be embedded in any interdisciplinary engineering work (51, 52).

To this end, the approach used in this pre-prototype was validated during multiple meetings with a therapy expert. In the present case the expert has a BSc in Occupational Therapy, MSc in elderly physical activity and Ph.D. in Sports Science, in Biomechanics area, is the Coordinator Professor in the Health School of the Polytechnique Institute of Porto, Director of its Clinic opened to the city of Porto community, and with a long experience in Hospitals' rehabilitation clinics in the same city. During the meetings, several topics were analysed to foresee the pre-prototype's core functionalities' usefulness in neurological or orthopedic cases.

According to the therapy expert, the need for realism in scene augmentation is less critical in orthopaedic cases since for them there is no alteration in the central nervous system. Nevertheless, the gamification process, even for this type of patient, may play an important role as it typically contributes to the immersion level of the therapeutic sessions. In the therapeutic context, a playful environment is considered to may have a positive impact by transforming a boring activity into an enjoyable, challenging one.

Also, there is the feeling that the proposed system will offer interesting benefits in exercises aiming at increasing movement amplitude, adjustments to the arm strength and the possibility of tuning patients' anthropometry, due to the system's flexibility on patient customization and progress, following the treatment evolution. Yet, the possibility of having the entire routine of exercises easily programmed by a therapist, by simply using a user-friendly interface to provide the patient with activity autonomy, is another advantage pointed out, allowing therapists to supervise more than one patient at a time.

### 2.2. The pre-prototype

The presented pre-prototype aims to explore the possibilities of using a AR application and a collaborative robot to stimulate visual, audio, and haptic senses to promote user engagement in the rehabilitation process. The two main components of the system are the collaborative robot, used to create haptic stimulation, and as games' controller. MH_2 is used to create an augmented reality interface, through which the user can interact with the system, select the robot's actions, and also serves to display additional information regarding the robot's actions, for example, the path which the robot will follow or the path the user will need to perform during the game.

Since collaborative robots can operate in the same space as humans without safety concerns, their use can help in different stages of rehabilitation by either guiding the users' movement in the initial stage or later creating a resistive force to the users' activity. In the pre-prototype's current version, the robot can guide the user or act as a joystick that does not resist the user's imposed force. These operation methods can be accessed by the AR interface, which supplements the robot operation with a virtual visualization of the robot's motion or the path the user will need to follow in the game setting.

**Figure 1:**
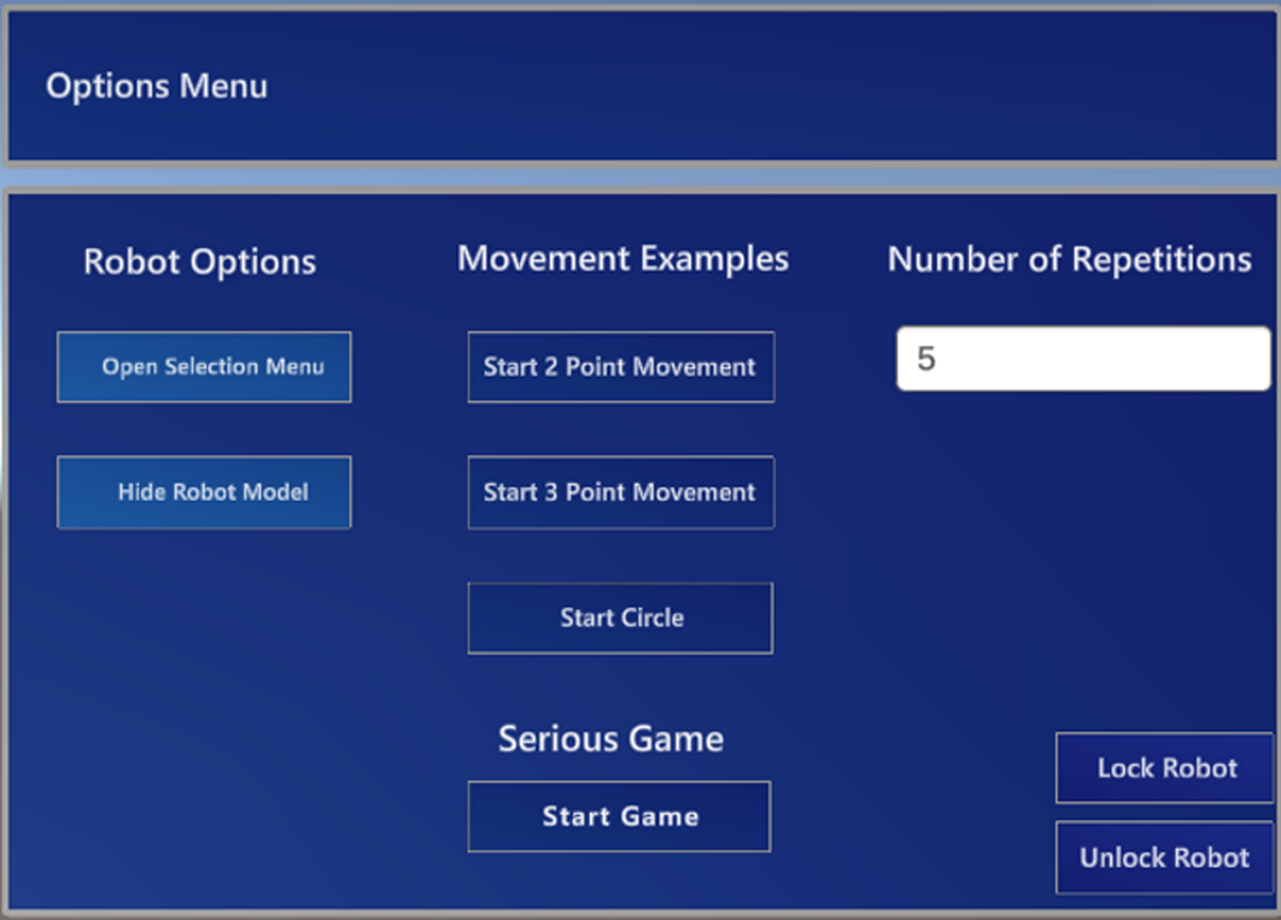
Menu for the training phase.

**Figure 2:**
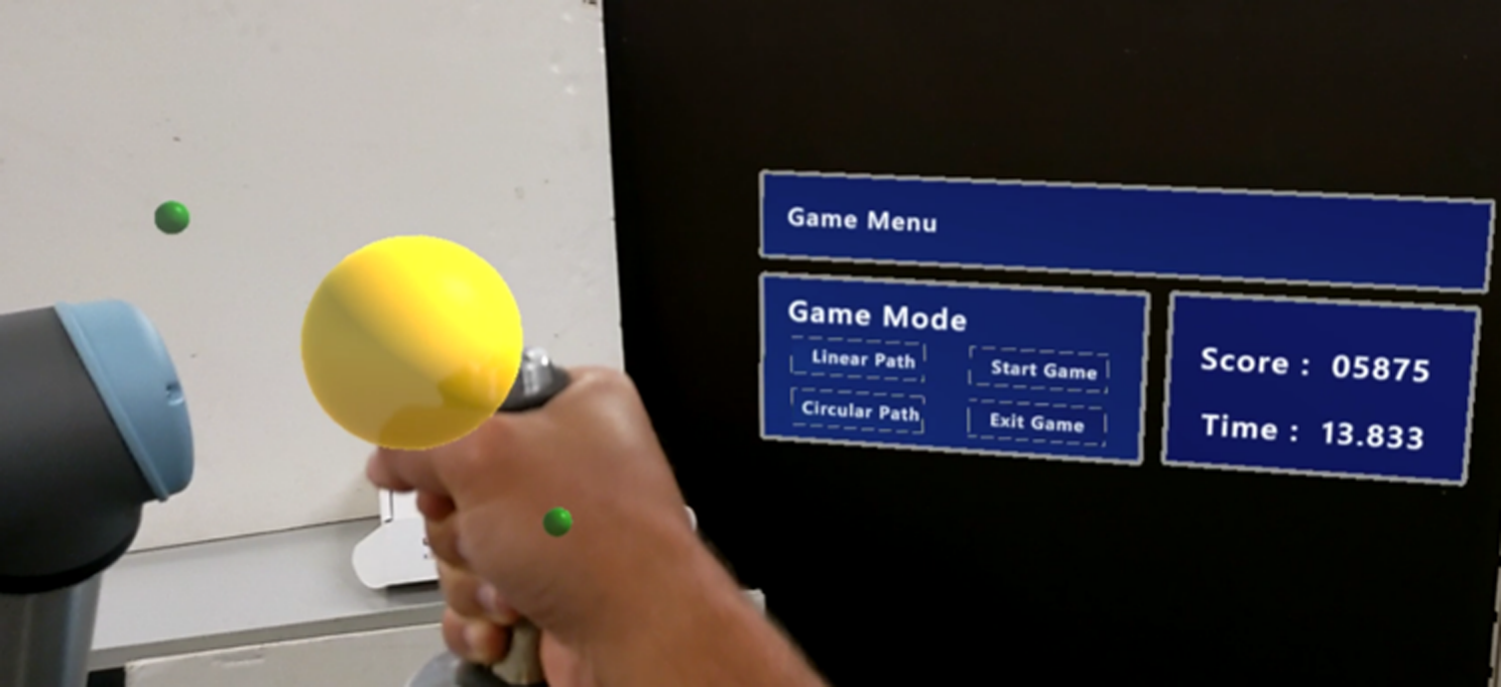
Participant prespective while using the system.

**Figure 3:**
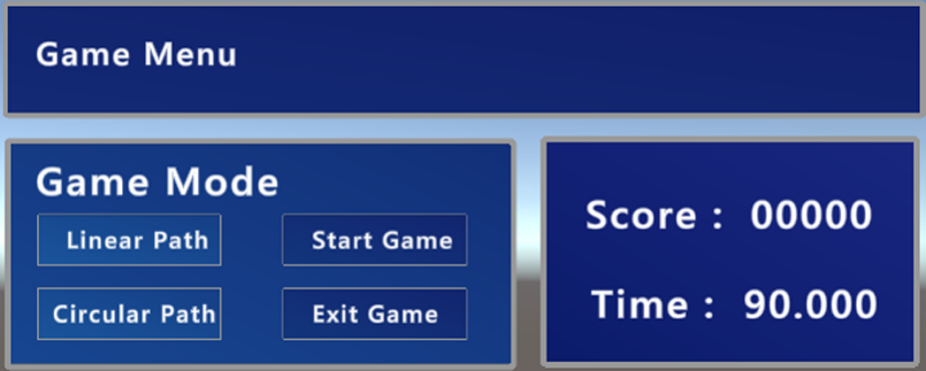
Game menu.

The interface presented to the user is divided into two different menus. In the first menu (see Figure 1), it is possible to interact with the robot by pressing the top three buttons in the middle column. These correspond to three predefined paths the robot will perform autonomously, with small green spheres appearing as helpers (as seen in Figure 2), so the user will know what motion the robot will make. On the right-side column, the user can change the number of repetitions the robot will perform for the previously selected movement. By pressing the “Start Game” button, the menu will disappear, and the game menu (see Figure 3) will be visible. In this menu on the left-side column, it is possible to choose which path to use during the game, and by pressing the “Start Game” button, the game will start. When the game starts, the time will start counting down, and the current score will be displayed next to the “Score” text. The game menu will close by pressing the “Exit Game” button, and the first menu will be shown.

### 2.3. Software tools and development

**Figure 4:**
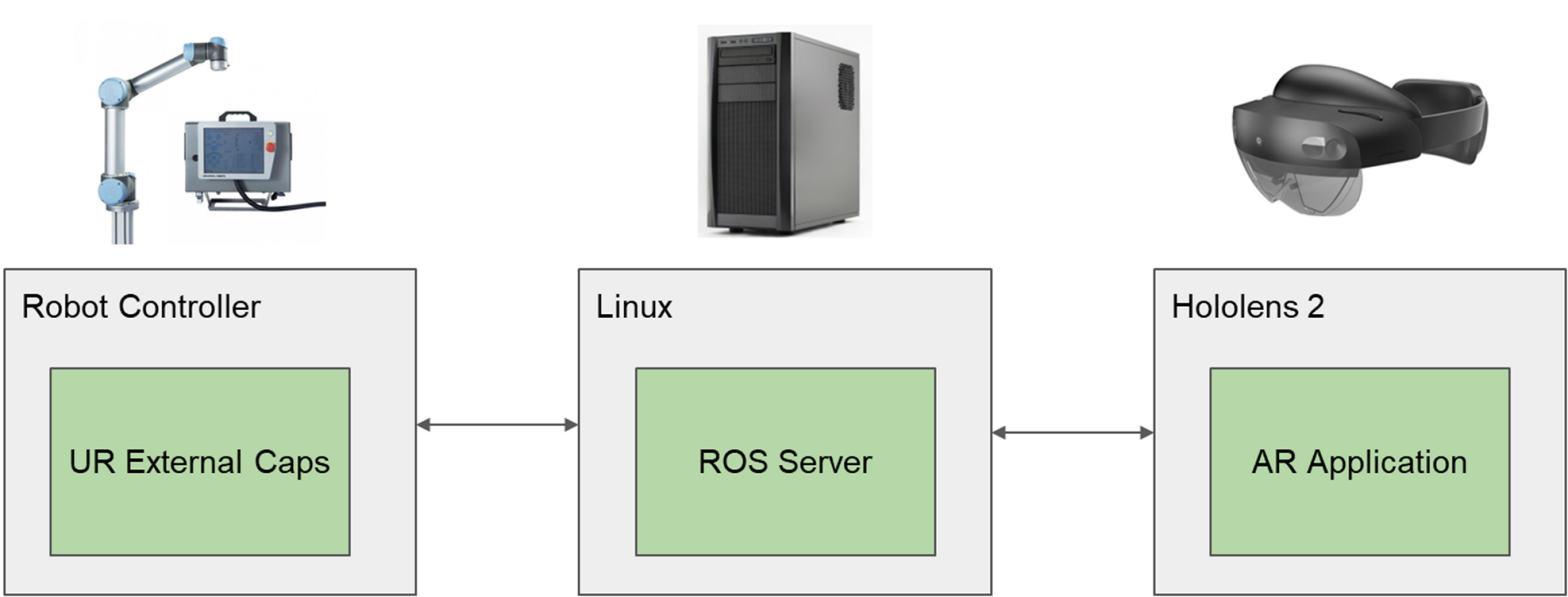
System architecture diagram.

The system includes a personal computer running Linux with mid-range hardware, a UR5e collaborative robot, and the MH_2. The application running on the HMD was developed based on the Unity game engine. The system's architecture is shown in Figure 4.

The Linux machine is running a ROS server, to which the AR application and the robotic arm connect, enabling the control of the robotic arm in response to commands sent from the application running on the HMD. The robot control was done with two different approaches. In one of the control approaches, the application running on the HMD sends the points that portray the selected default motion path to the server. The server then sends these points to the robotic arm as position references. The other control approach mode is based on velocity control, which is only used in a serious game option. In this control, the user grips the handle in the free extremity of the robot and depending on the direction of the force applied to the handle, the robot then moves in the same direction. With this latter approach, it is possible to have the robot act as a haptic device that can either help the user perform the movement or stimulate the user by creating a resistive force to the user's movement. With this capability, the system can be used in all stages of rehabilitation, starting with helping the patient relearn upper-limb movements by guiding him to a later phase that requires intensification of muscular strength.

### 2.4. Pre-prototype case study

#### 2.4.1. Description of the case study

The study consisted of two steps: using the system and answering a questionnaire. The participants were asked to perform a defined task with the system and answer a questionnaire concerning their experience. This questionnaire was composed of the following three different questionnaires: System Usability Scale (SUS) (53), initially created by John Brooke in 1986 to support a subjective measure of the perceived usability of a system; the User Experience Questionnaire—Short Version (UEQ-S) (54), which is an end-user questionnaire to measure user experience; and Flow Short Scale (FSS) (55, 56) for measuring the flow, challenge, and anxiety induced by the experience.

Before the activity started, participants were given instructions on using the system and their goals. After an initial explanation, the participants were asked to wear the MH_2. Then they had an exploration training period of 5 min before being asked to perform the designated tasks. Participants' questions or difficulties during the exploration phase were answered and explained to help them achieve the desired interactions with the system. After the initial 5 min of exploration, participants were asked to perform the experiment, which consisted of playing two simple, serious games. When each participant concluded the experience, they were then asked to answer anonumously the questionnaire available online.

Additional information was gathered by the first author during the participantś practical use and system exploration. It was based on their questions, comments and behaviour. This methodology was followed during all phases of each participant trial.

#### 2.4.2. Methodology and sample characterization

A cross-sectional study to evaluate the system usability was conducted using a non-probabilistic sample of 31 individuals (42% women and 58% men; 13 participants between 21 and 30 years old, 10 participants between 31 and 40 years old, and 8 participants between 41 and 60 years old). The participants' education level distribution of the sample, according to the ISCED2012 classification, is: L3 [1], L6 [5], L7 [9], and L8 [16]. None of the participants had previous experience with MH_2 or with a collaborative robot and had no past experience in physical rehabilitation. All the participants stood up during the usage of the system. Participants volunteered after receiving information sent by email. The first author carried out data collection in May 2022. The questionnaire was implemented in google forms, with no authentication required to guarantee anonymity. Informed consent was given by the participants previously to the questionnaire, and they were free to refuse this step. All participants agreed to fill out the questionnaire.

During the training phase, participants were encouraged to experiment, press virtual buttons, move virtual objects and observe how the robot would react to their commands. The main goal of this training phase was to have the participants adapt to how to interact with the virtual objects using the MH_2 interaction techniques.

**Figure 5:**
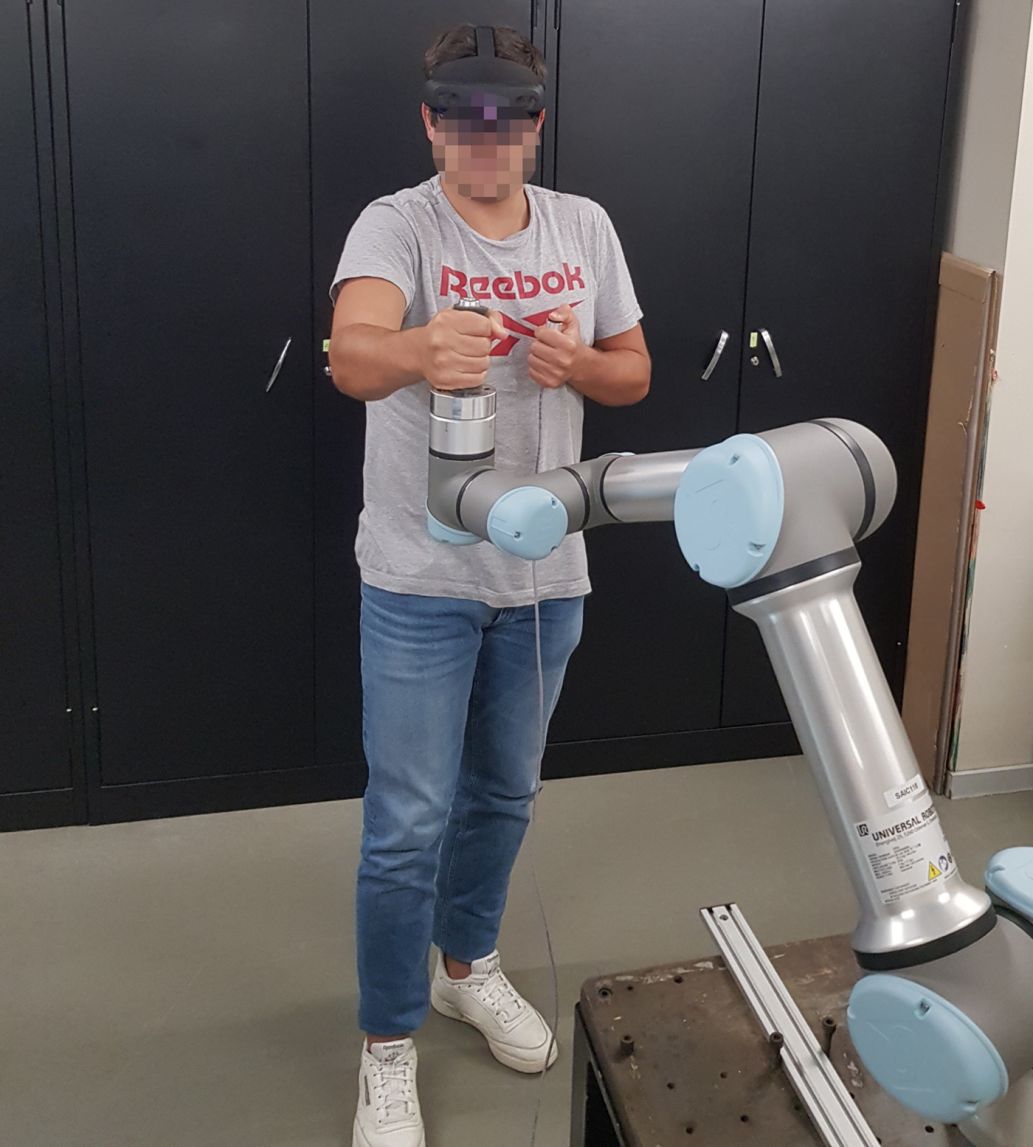
Participant using the system.

Once the training phase was over, participants were asked to press the “start game” button to change the menu displayed to the game menu. Then, they were asked to play the serious game twice during the evaluation phase, using linear and circular paths. The sequence of each path to use was the user's choice. The game lasted 90 s for each path, and the objective was to score as many points as possible. The user commanded the robot with one hand placed on the tool point and needed to follow the presented path to play the game. The faster the user got to the next point, the higher score he would get. Figure 5 shows a participant using the system, and Figure 2 shows their perspective.

The questionnaires presented to the participants after using the system were focused on the system interface usability to evaluate the idea of using augmented reality as an interactive interface that allows the command of a collaborative robot.

#### 2.4.3. User experience questionnaire—short version

The UEQ-S aims to evaluate the overall experience that the participants had while using the system and allows subdivisions of the evaluation into two qualities besides the overall evaluation: the pragmatic and hedonic qualities. To process the data from this questionnaire, there is available online (57) a script to help with data processing and also allows to compare the results obtained in the study with results of other stored studies of different areas. This provided file also allows for identifying inconsistencies in the answers, which can help identify participants' random or not serious answers. These inconsistencies are found by applying a simple heuristic. All items in a quality (Pragmatic or Hedonic) should measure a similar quality aspect by checking how much the best and worst evaluations of an item in a quality differ. If the difference is higher than three, this can be considered an indicator of a problematic data pattern.

#### 2.4.4. System usability scale

The system usability scale was developed by John Brooke in 1986 (53) and is the most used questionnaire for measuring perceptions of usability. SUS is composed of ten statements with responses ranging from 1 (strongly disagree) to 5 (strongly agree). A scoring system has been developed using the following procedure (58): subtracting one from the user responses to the odd-numbered items and subtracting the user responses from five to the even-numbered items. The resulting score will range from 0 to 100. This score is not a percentage. According to ref (59), based on almost 500 studies across various applications, the average score of 68 marks the 50% percentile. Even though SUS is a widely used metric for usability evaluation, it does not allow for the detection of usability problems. It only provides information on how users perceive the application's usability. Other metrics, such as completion time and task completion rates, should also be used. One of the significant benefits of using this questionnaire is that since it is technology independent, it can provide a reliable, valid, and quick measure of ease of use.

#### 2.4.5. Flow short scale

The Flow Short Scale measures the flow, anxiety, and challenges the users perceive while using a system. The flow state in psychology is the mental state in which a person performing an activity is fully immersed in a feeling of energized focus, full involvement, and enjoyment in the activity process. In comparison, anxiety is an emotion characterized by feelings of tension and worried thoughts that can come from possible fear of failure or making mistakes while performing a task. FSS uses fourteen statements with responses ranging from 1 (not at all) to 7 (very much). Only the last statement uses a response from 1 (too low) to 7 (too high). The first ten statements are directly tied to measuring the flow perceived by the users, eleven to thirteen statements to the anxiety, and the last statement to the challenge. The values for the user's perceived experience can be calculated on a scale from 1 to 7 for each component (60). On this scale, a number four is considered a neutral value, and higher or lower values can mean a positive or negative evaluation.

As there is not a validated Portuguese version of the FSS scale (FSS scale V_PT_), the used version was worked by the first author and double-checked by the other two authors. The participants did not report any misunderstanding or ambiguity issue.

## 3. Results and discussion

This section presents the questionnaire's results and discussion. Since the three questionnaires aim to evaluate different aspects of usability and interaction, their results and discussion will be separated into subsections.

### 3.1. User experience questionnaire—short version

With the data collected, the first step was to check if there were any inconsistencies with the participant's answers. To that end, the heuristic previously described was applied, and only two answers were flagged as possible suspicious data, which were considered to be removed from the data. The detected inconsistencies were observed in the answers of two participants, which were found for only one of the qualities (Pragmatic or Hedonic). If an answer only presents inconsistencies for one of the qualities, it is not considered problematic. Therefore, the inconsistencies found were not considered problematic. Table 1 shows the overall results from the UEQ-S, where each column shows the number of participants who answered the corresponding number.

**Table 1:** Summary of user experience questionnaire results.

Item		Scale								Qualities
		1	2	3	4	5	6	7		
1	Obstructive	0	0	0	5	9	12	5	Supportive	Pragmatic Quality
2	Complicated	0	1	1	3	7	15	4	Easy	
3	Inefficient	0	0	0	3	7	13	8	Efficient	
4	Confusing	0	0	1	5	4	14	7	Clear	
5	Boring	0	1	0	3	6	7	14	Exciting	Hedonic Quality
6	Not Interesting	0	1	0	2	3	10	15	Interesting	
7	Conventional	0	1	0	1	4	9	16	Inventive	
8	Usual	0	0	0	2	3	5	11	Leading-edge	
		−3	−2	−1	0	1	2	3		

**Figure 6:**
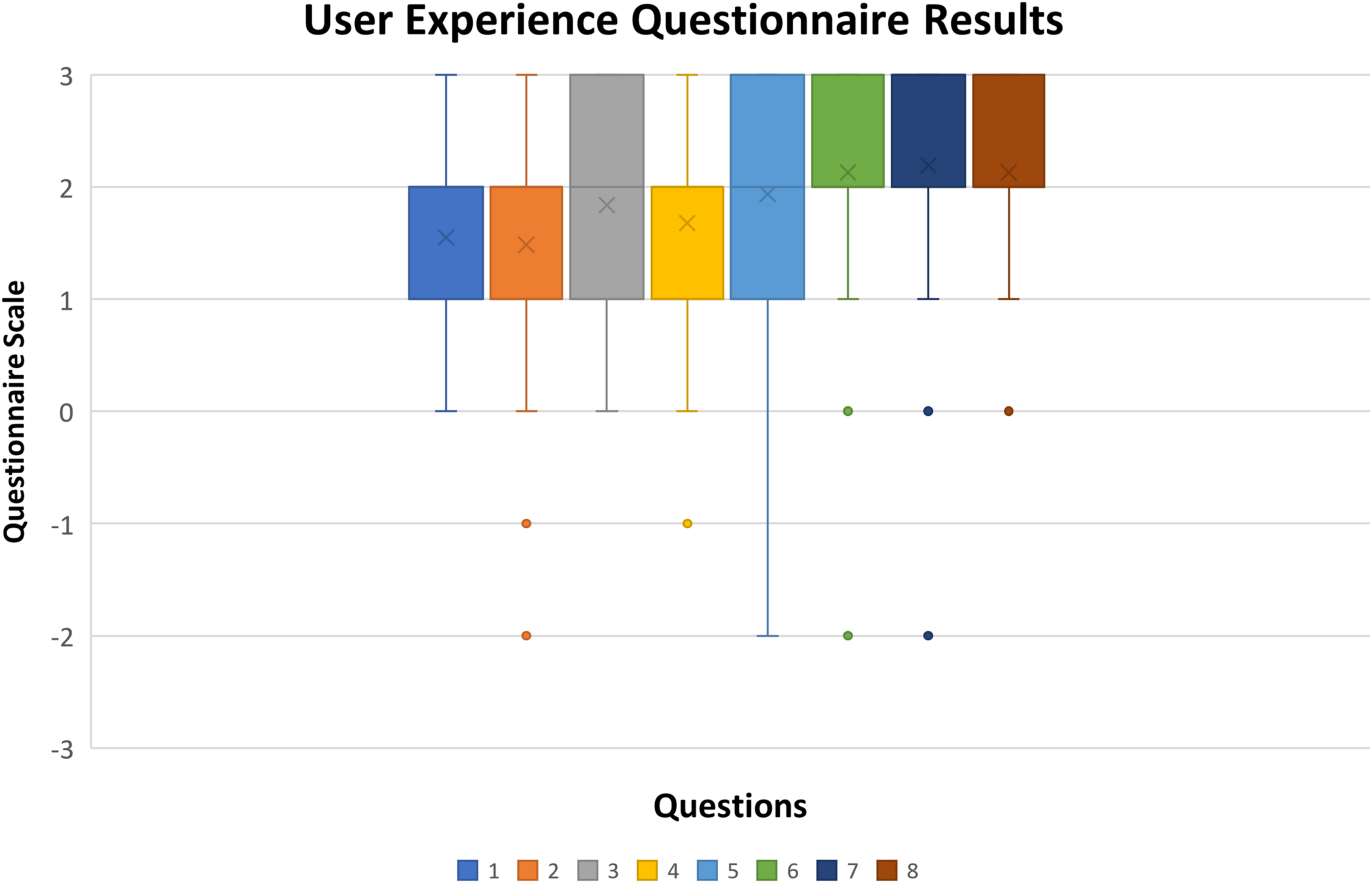
Boxplot chart of user experience questionnaire results (x stands for mean value, rectangle upper and lower quartile with at least 50% of values, whiskers represent the 0th and fourth quartile when there are values).

By applying a data transformation to the numbered options, the neutral answer becomes zero instead of four. This data transformation allows for easier reading of the values for the range of the scales, as a minus three represents a bad value and a plus three a good one. Figure 6 shows each item's results variation as a boxplot chart.

**Figure 7:**
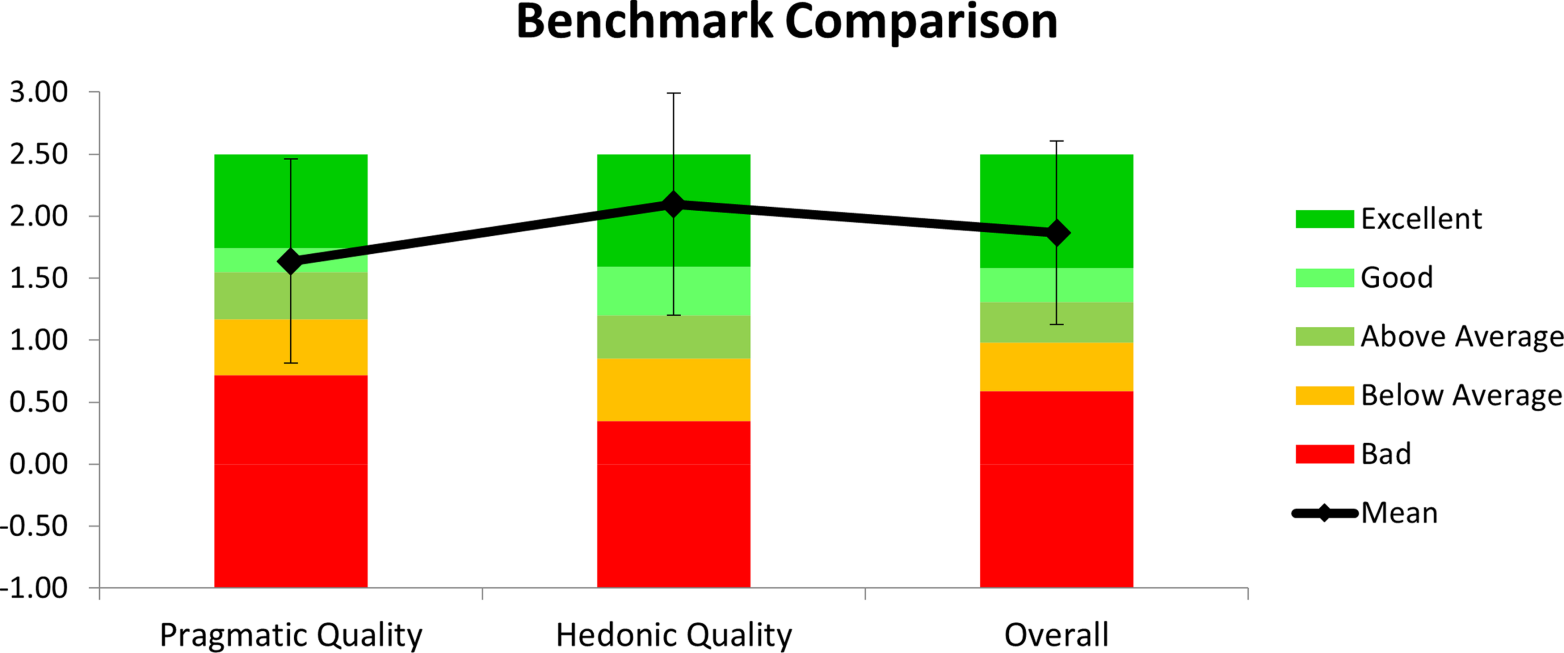
User experience questionnaire qualities results and benchmark.

In all items, it is possible to observe a clear tendency in the majority of answers towards positive evaluations as the mean values for each item are high, considering an interval of [−3, 3]. In most items, 100% of the answers are between [0,3], with at least 50% being higher than 1. The results show that most participants agreed on the evaluation items for the UEQ-S. According to (61), the first four items have a higher factor in the pragmatic quality evaluation and the last four in the hedonic quality evaluation. In the present study, Figure 7 represents a comparison of its results within the database of 468 different studies (of distinct products that used the UEQ as an evaluation method). For this purpose, the quality values (Pragmatic and Hedonic) are obtained by calculating the mean value of the means of the corresponding items (Table 1 lines), which can be seen in Figure 7.

The values shown indicate the reported user's experience during participation. Considering an interval of [−3,3], the mean values for each scale and overall evaluation show that participants positively evaluated the experience and its usage.

According to the data and by comparison with the database offered by the UEQ, the hedonic quality reached an excellent rating. However, the usability of the system that is directly related to the pragmatic quality only obtained a good rating. These suggest that a special care should be taken in consideration for usability in the next development step. This issue can be overcome by enabling exercise user customization which reflects on robot parametrization and is also important to AR field of view, and game dynamics [for example, movement amplitude (user height, limb length, shoulder angles), speed, active or passive mode, game challenge level, …].

### 3.2. System usability scale

System Usability Scale (SUS) questionnaire aims to evaluate the perceived usability of our system. Participants were asked to fill out the questionnaire after using the system. Table 2 shows a summary of the collected data.

**Table 2:** Summary of system usability scale results.

Item		Strongly Disagree				Strongly Agree	*N*
		1	2	3	4	5	
1	I think that I would like to use this system frequently	0	0	8	14	9	31
2	I found the system unnecessarily complex	10	15	6	0	0	31
3	I thought the system was easy to use	1	2	5	13	10	31
4	I think that I would need the support of a technical person to be able to use this system	7	5	8	9	2	31
5	I found the various functions in this system were well integrated	0	0	4	16	11	31
6	I thought there was too much inconsistency in this system	9	18	3	1	0	31
7	I would imagine that most people would learn to use this system very quickly	0	0	5	10	16	31
8	I found the system very cumbersome to use	18	11	1	1	0	31
9	I felt very confident using the system	0	2	9	7	13	31
10	I needed to learn a lot of things before I could get going with this system	19	10	2	0	0	31

In the SUS questionnaire answers can be assumed as positive if the user strongly agrees or strongly disagrees, depending on each particular statement. The results, on Table 2 (see columns 1 and 5), show that participants exhibit high agreement on their answers, except for items 3 and 4. These two items highly depend on how much each individual can learn and adapt to new technologies. As none of the participants had previous experience with the system components, in particular using MH_2, some participants felt they would need a technical person to guide them to use the system. The SUS scores were calculated using the participants' answers. Table 3 shows the obtained SUS score.

**Table 3:** Obtained score of system usability scale.

Score				
Mean	Median	Q1	Q3	IQR
78	80	70	87.5	17.5

**Figure 8:**
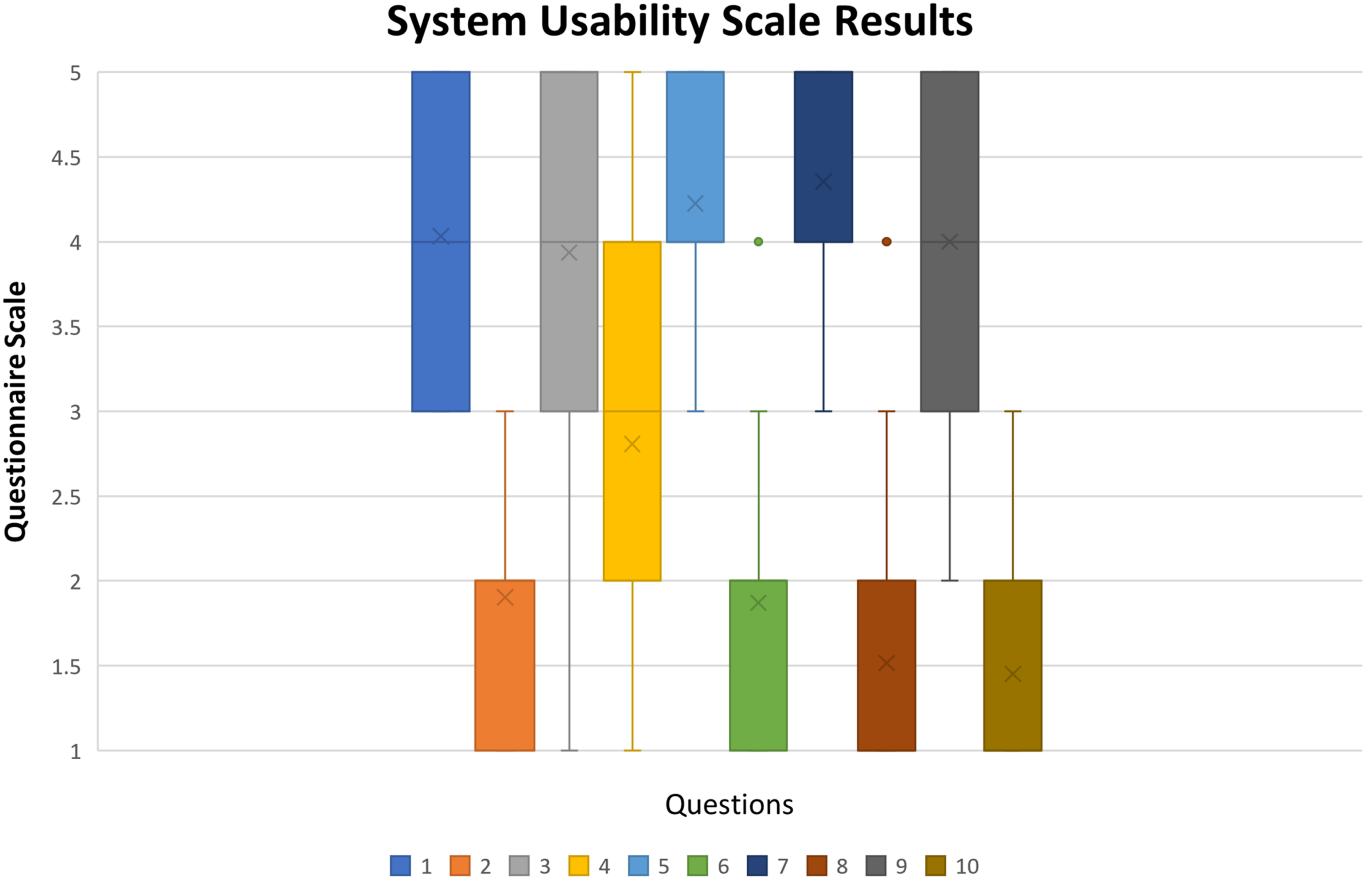
Boxplot chart of system usability scale results (x stands for mean value, rectangle upper and lower quartile with at least 50% of values, whiskers represent the 0th and fourth quartile when there are values).

The score was calculated for each participant's answer using the formula described in section 2.3.4., which shows a mean score of 78, which, according to (59), places the results in the 80–84 percentile range compared to other studies using SUS. This result can be seen as satisfactory as a first usability evaluation of the pre-prototype, encouraging the researchers to continue the concept development. Median value is 80 with a Q1 value of 70 and a Q3 value of 87.5, giving an interquartile range (IQR) of 17.5. The given interval is relatively small, considering that the range of the possible values is from 0 to 100. A boxplot chart of SUS results is shown in Figure 8.

In the SUS questionnaire answers, values can range from 1 to 5, where one is considered “Strongly Disagree” with the statement and 5 “Strongly Agree.” However, depending on the item, a higher or a lower value can be considered a positive or negative response. Items numbered with odd numbers have a positive evaluation if the participant agrees with the statement, and items numbered with even numbers have a positive evaluation if the participant disagrees with the statement. This is, items (1, 3, 5, 7, 9) have a positive evaluation if the answer is closer to 5, and items (2, 4, 6, 8, 10) have a positive evaluation if the answer is closer to 1.

Most of the items were evaluated positively by the participants. In item 4, answers were spread along the scale with a higher concentration between 2nd and 4th levels. This results in a neutral evaluation of item 4, as shown in Figure 8. This inconsistency in answers to item 4 indicates that improvements must be made to create a more intuitive interface. On the other items, it is possible to see a clear tendency in the participant's answers, as most of the values are encouraging concentrated on one end of the scale. The problem of item 4 will be partially solved by the customization referred in the last paragraph of the section 3.1. Additionally, creating warning signals when there are virtual objects out of the field of view, will easily tell the user that he/she should move their heads in the direction of the warning signal. With those improvements the authors believe that the technical person will not be required to help as it is reflected in item 4.

### 3.3. Flow short scale

Flow Short Scale (FSS) questionnaire aims to evaluate the perceived flow, anxiety, and challenge of using our system. Participants were asked to fill out the questionnaire after using the system, using a scale from 1 (not at all) to 7 (very much). Table 4 shows a summary of the collected data.

**Table 4:** Summary of flow short scale results.

Item			Scale							
			1	2	3	4	5	6	7	
1	I feel just the right amount of challenge	Not at all	1	2	2	6	12	6	2	Very Much
2	My thoughts/activities run fluidly and smoothly	Not at all	0	0	1	3	7	14	6	Very Much
3	I don't notice time passing	Not at all	2	2	3	3	7	4	10	Very Much
4	I have no difficulty concentrating	Not at all	5	6	2	1	4	7	6	Very Much
5	My mind is completely clear	Not at all	0	0	0	1	8	13	9	Very Much
6	I am totally absorbed in what I am doing	Not at all	0	0	0	1	2	13	15	Very Much
7	The right thoughts/movements occur of their own accord	Not at all	0	0	2	1	8	9	11	Very Much
8	I know what I have to do each step of the way	Not at all	0	0	2	0	6	15	8	Very Much
9	I feel that I have everything under control	Not at all	0	1	3	4	5	10	8	Very Much
10	I am completely lost in thought	Not at all	16	10	2	0	2	1	0	Very Much
11	Something important to me is at stake here	Not at all	2	2	5	8	3	8	3	Very Much
12	I won't make any mistake here	Not at all	4	4	5	8	6	3	1	Very Much
13	I am worried about failing	Not at all	9	5	3	5	2	6	1	Very Much
14	For me personally, the current demands are …	Too Low	1	1	6	11	10	2	0	Too High

**Figure 9:**
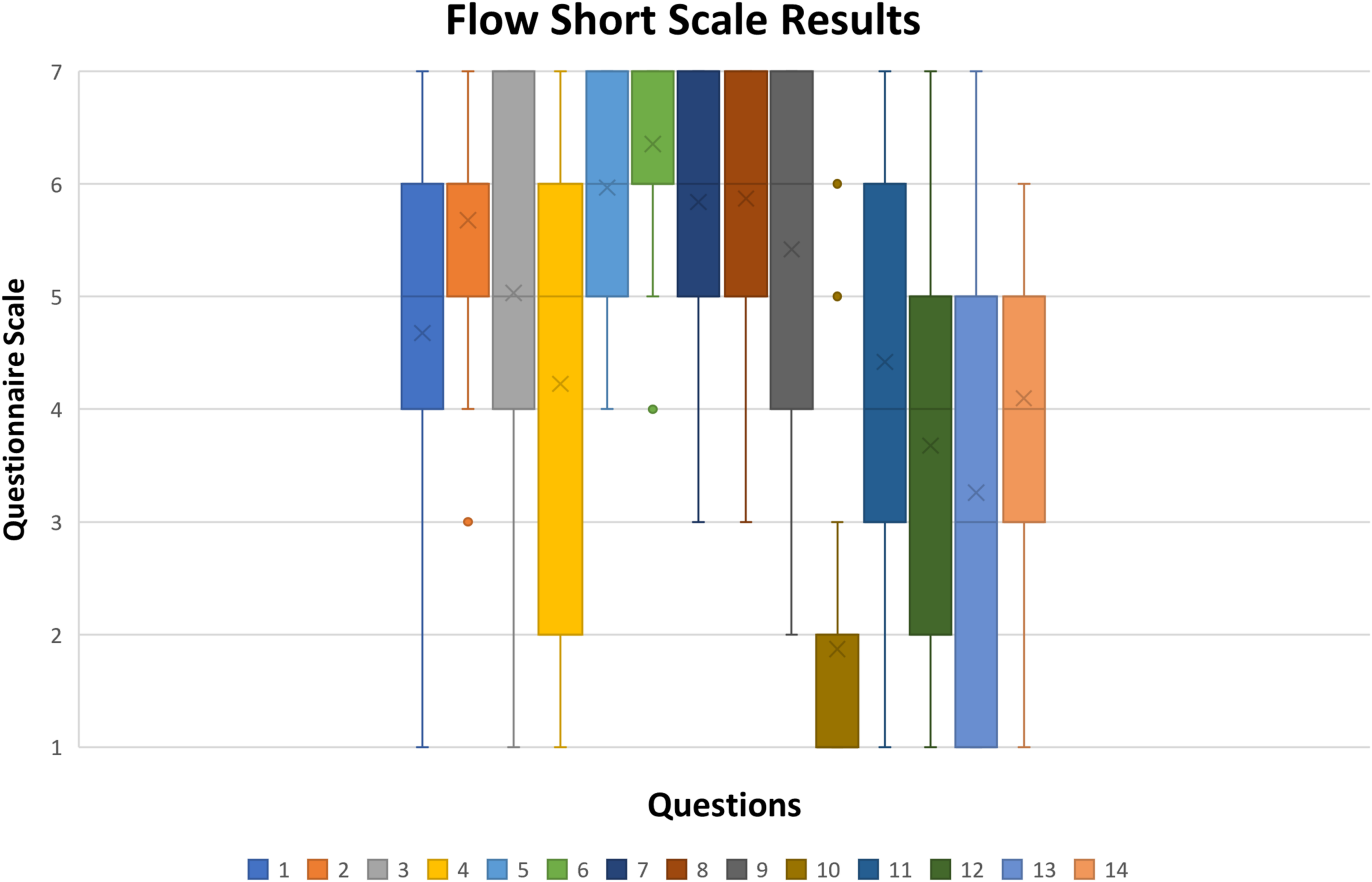
Boxplot chart of flow short scale results (x stands for mean value, rectangle upper and lower quartile with at least 50% of values, whiskers represent the 0th and fourth quartile when there are values).

As it is possible to be observed, the answers to most of the items of this questionnaire do not have a clear tendency to one of the extremities as this questionnaire evaluates each individual psychological factor while using our system. Boxplot chart of Flow Short Scale results in Figure 9 shows each questionnaire item's results variation.

**Figure 10:**
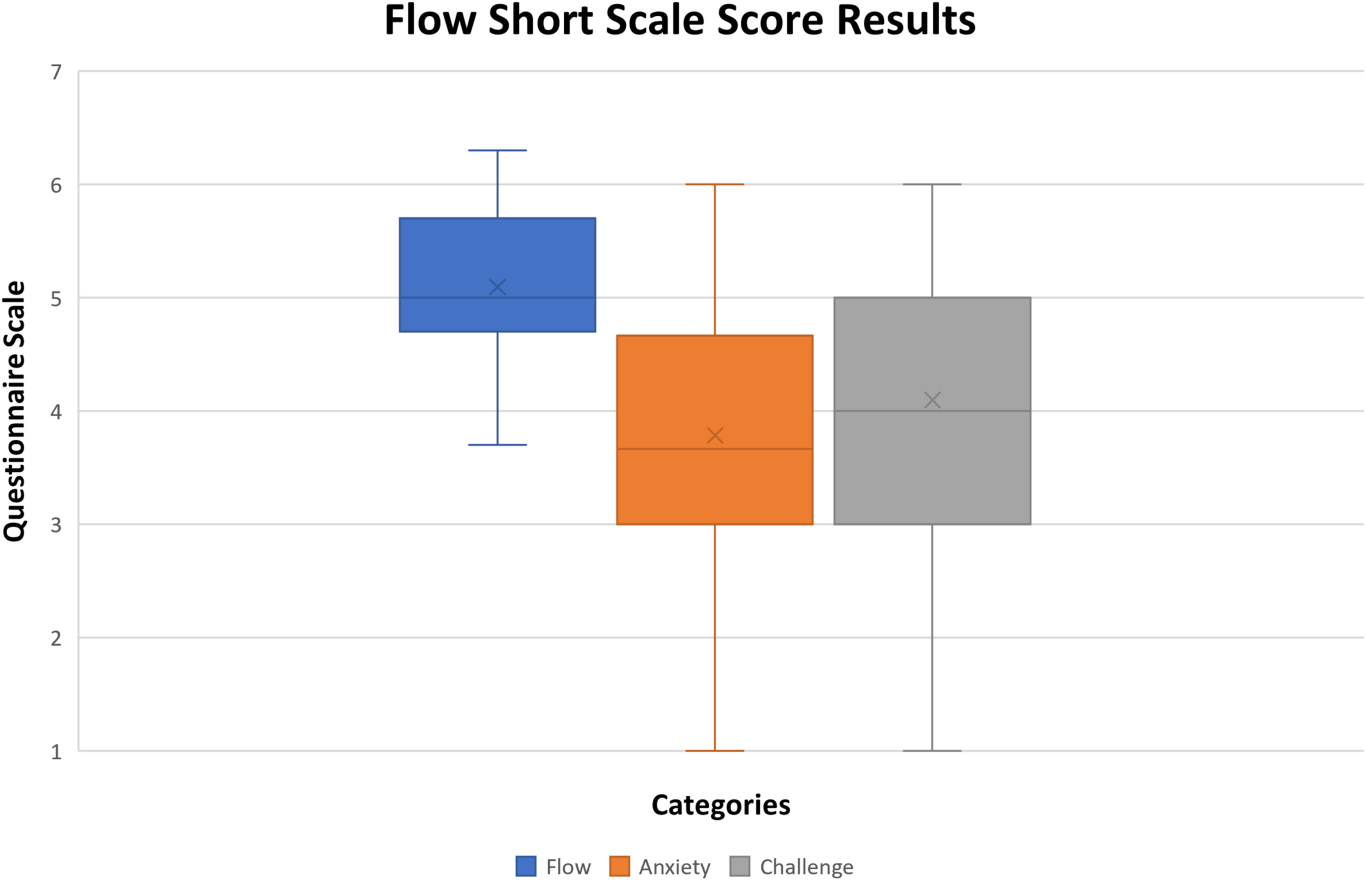
Boxplot chart of flow short scale score results (x stands for mean value, rectangle upper and lower quartile with at least 50% of values, whiskers represent the 0th and fourth quartile when there are values).

Quartiles 2 and 3 are represented by the box and contain 50% of the data. In most items, their range is either 2 or 3 values, and in some cases, like items 4 and 13, their range is four. This indicates that participants' answers were spread in the range of possible answers. This result of the perceived user experience can be heavily impacted by personality traits or past experiences in life, as some people are afraid of making mistakes or failing a task, and others do not. Since the presented version to the participants was translated by the researcher and was not validated, some questions might not have got the most accurate form; by comparing items answers 10, 5, and 6, it is possible to see opposite results. Alternatively, what one person considers uninteresting, others can consider entertaining. In order to better understand the results, the scoring system provided by FSS needs was applied. To calculate the scoring system, an online tool is currently available by the questionnaire author (60). Each participant's answer was considered, and the summary of the scores obtained is in Figure 10.

The resulting scores obtained from the questionnaire are divided into flow, anxiety, and challenge. The values obtained in this first evaluation of the pre-prototype were very satisfying.

The flow variable is the mental state in which a person performing some activity is fully immersed in a feeling of energized focus, full involvement, and enjoyment in the process of the activity. Its positive feedback with a median value of 5, 4.7 for Q1, 5.6 for Q3 and 0.9 for IQR encourage to continue the development of the concept, considering the scale ranges from 1 to 7. Also, the reached score has been good, the authors believe that future improvements described in the previous sections will help in improving the flow category (in fact, they will improve engagement, usability, and make the system usage more intuitive/clear). During rehabilitation exercises patients are usually seated, therefore in later system test evaluations participants should be also seated.

The anxiety perceived by the users when using the system was also satisfying since the median value of 3.7, 3.2 for Q1, 4.5 for Q3 and 1.3 for IQR, which places most of the results approximately to the middle of the scale. The anxiety value characterizes the feeling of tension and worrying thoughts that occur during the process of using the system. Considering the value is clearly in the middle of the scale, avoiding stress caused by falling the game objectives will help achieving a better score (lower score). The cause for this result is clearly related to the identified problems along with the analyses already reported in the previous sections.

Item 14 of the questionnaire is the only item directly tied to the challenge evaluation of the system. Looking at the scale of the paper version of the questionnaire (62), the scale middle point is signalled as “just right.” Therefore, being 4 the median result obtained, can be considered the “just right” result, with 3.5 for Q1, 5 for Q3 and 1.5 for IQR. This is a very good result that can be expected for the perceived challenge of a preliminary game pre-prototype, which encourages further development.

### 3.4. Learned lessons and limitations

From the set of tests implemented and analysed, as well as information gathered by the first author following carefully the participants' use of the system, some issues that may have influenced negatively the obtained indicators of the system's usability have been identified: the relative position between the robot base and the participants' eye level (participants had to stand up during the system usage and the robot base was at a fixed position), and possible participant vision impairment in any of his/her eyes.

In fact, in some cases, differences in the relative position were causing the system more problems in identifying the users' hand: the hand detection was done using the MH_2 cameras, so the relative position of each user's head (eyes) to the topmost and lowest points of game paths were different, triggering differently the hand orientation while grabbing the free handle of the robot. Therefore, this impacted how well the system was able to identify and track the participants' hands. In the present case, shorter participants had more difficulties using the system, especially when the path was on its topmost points.

The second issue observed, was due to the fact that one of the participants had one eye with vision impairment. This factor came to our knowledge in a later comment and was not considered previously in the study. Due to the participants' anonymity, it was impossible to remove the respective answers. As this is 1 element on a sample of 31, the corresponding answer was considered not to imply a significant bias on the results, acting more as an outlier. Nevertheless, this type of visual impairment should be considered in future conditions for selecting participants.

The evaluation presented even with healthy participants, and the discussions with the therapy expert were essential to determine if the system usability was done properly, or if new approaches should be taken.

Therefore, even though, the proposed system is composed of various commercial devices, an initial usability evaluation of the system interface was needed so it would be possible to answer to (1) do users found the system friendly to use or too complex? (2) do they can use the system without guidance, or if revisions are needed so no guidance is needed? Considering the results of the different questionnaires, we can conclude that users found the system easy to use, but some needed help form a technical person to successfully conclude the tests. These results indicate that some limitations on the implementation of the system need to be corrected as described above to have a system that can be used without guidance.

From the researcher observation during the user's participation in the trial, we can infer that the MH_2 is suitable to be used in this application, and also that the combination of the tactile feedback provided by the robot and the visual aid provided by the MH_2 can successfully be used as a tool to help participants perform the correct movements.

However, the perceived limitations have shown that attention needs to be dedicated to the hand tracking process, the inclusion of different input functionalities during exercises design application in order to optimize the field of view limitation of the hardware. Finally, next sample should exclude any participant with a visual impairment situation.

Also, it should be said that although only one highly qualified expert was involved in the assessment of the user interface, future work of higher order complexity will need to be assessed by higher number of therapy experts and physiatrists. It will be fundamental to ensure the adequacy and effectiveness of the future application developments to follow correct therapy protocols.

The results of this pilot study showed that some revisions are needed but the core idea was well accepted by the users which encourages further development.

## 4. Conclusions

This paper presents and discusses a concept of an interactive system aiming for its use as an upper-limb rehabilitation supporting tool. Given that low motivation and lack of adherence during the rehabilitation process can be significant factors that can lead to abandonment or unsuccessful treatment. To this end, the proposed system uses augmented reality and a collaborative robot, enabling stimulation of various senses, mainly auditory, visual, and tactile, to promote patient retention. These stimuli enable an engaging environment and help promote positive emotional triggers that lead to an enjoyable experience. A pre-prototype was developed to validate the core idea's usability using the Microsoft HoloLens 2 and UR5e collaborative robot. A preliminary usability study is reported. For this evaluation, a cross-sectional study using a non-probabilistic sample of 31 individuals based on the developed augmented reality interface to interact and control a collaborative robot is presented and discussed. Participants were asked to use the system and then answer a questionnaire to obtain data regarding the system interface usability and how the integration of the different technologies could be a factor in success. The questionnaire was composed of three standard questionnaires: System Usability Scale, User Experience Questionnaire, and Flow Short Scale. The results obtained were positive, for the system usability scale, the obtained results place it in the 80 to 84 percentiles in comparison to the literature. For the User Experience Questionnaire, the results obtained for the pragmatic, hedonic and overall qualities compared to the benchmark give a good or excellent evaluation of the system by the participants. For the Flow Short Scale, the results obtained for the flow and challenge components perceived by the system users were also positive. These outputs encourage further development of the system. Observation done by the researcher performing the study during participants' experimentation revealed that some changes need to be implemented to improve system usability. For example, customizing the Robot tool position relative to the user's height to help improve usability. The proposed system evaluated on the perspective offered by the user interface was presented and explored to a therapy expert in order to have her appraisal of its adequacy and usefulness towards rehabilitation. During this discussion, different additional features such as system passive and active working modes, and the recording capacity of different parameters, among others, were also debated. Otherwise, the main idea of this study should have been abandoned. The comments were very positive and encouraging for using the system in upper limb rehabilitation, as it was considered to offer the potential to support therapeutic exercises typically executed using non-robotic devices. Moreover, the expert also considered the latent possibility of its easy customization based on the interface's ability to represent different rehabilitation apparatus and to tune parameters such as guidance, stiffness, and velocity, some relevant patient anthropometric characteristics, among others. Therefore, the expert assessment of the system was very positive and encourage future studies.

In terms of non-expert participants within different age categories, they reported little to no difficulties using the pre-prototype after the initial explanation and respective exploratory period. Participants also revealed that they would like to use such a system again in the future which can be seen as a motivational success. But still, some of the identified problems need to be corrected in future evaluations, such as, be possible to customize the exercises to each user, hand tracking (using the most efficient solution between, MH_2 hand tracking, or real time data from the robot free handle position). Exercise customization will also enable robot parametrization which can help in different aspects of usability (for example, movement amplitude, speed, active or passive mode, game challenge level, …). Additionally, warning signals may also be created to help users know if there are virtual objects outside their current field of view. Furthermore, in future evaluations any type of user impairment level, either visual or auditory, might be considered in order to verify if this can be a potential barrier to its use.

## Data Availability

The raw data supporting the conclusions of this article will be made available by the authors, without undue reservation.
